# Extracellular vesicles: Antiviral functions and applications in therapeutics and vaccines

**DOI:** 10.1016/j.isci.2025.113542

**Published:** 2025-09-10

**Authors:** Qing Gao, Yuqing Zhan, Jianhao Zhang, Dongyu Sun, Huayuan Xiang, Chenxuan Bao, Yuting Xu, Qianqian Gao, Lingxiang Mao

**Affiliations:** 1 Department of Laboratory Medicine, Affiliated Kunshan Hospital of Jiangsu University, Kunshan, Jiangsu, China; 2 Jiangsu Key Laboratory of Medical Science and Laboratory Medicine, School of Medicine, Jiangsu University, Zhenjiang, Jiangsu, China

**Keywords:** Immunology, Cell biology

## Abstract

Extracellular vesicles (EVs) are nanoscale membranous vesicles secreted by nearly all cell types, exhibiting dual regulatory mechanisms during viral infection: they facilitate infection spread by packaging whole infectious viral particles or functional viral genomes while concurrently delivering immunoactive substances to participate in host antiviral immune responses. Additionally, EVs themselves can exert direct antiviral effects, such as by competing with virions for binding to host cell receptors such as phosphatidylserine (PS) receptor. As natural nanocarriers, EVs have emerged as novel therapeutic delivery vehicles owing to their superior biocompatibility, low immunogenicity, and efficient penetration across physiological barriers. Through engineering strategies such as surface modification and cargo loading, EVs enable targeted delivery of therapeutic agents, including antiviral molecules and gene-editing tools, effectively suppressing viral replication and enhancing host immune responses. Furthermore, their unique antigen-presenting mechanisms and immunostimulatory properties underscore significant potential in vaccine development.

## Graphical abstract

**Figure:**
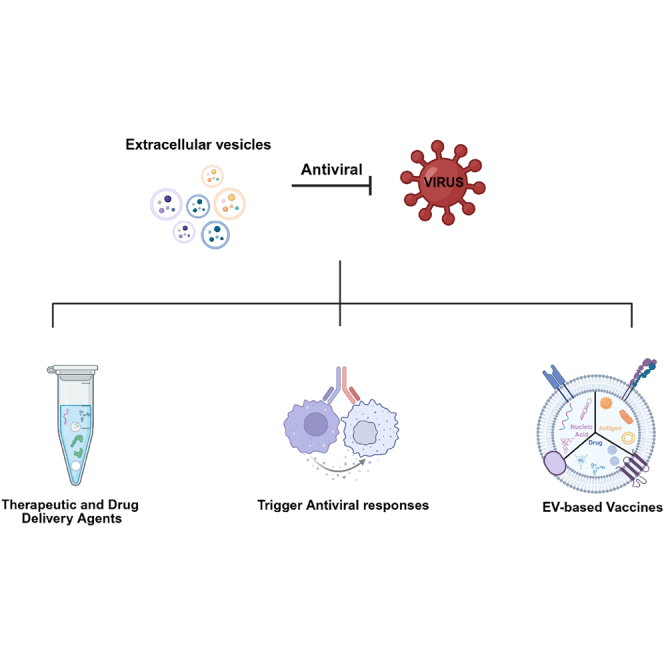

## Introduction

Extracellular vesicles (EVs) represent heterogeneous collections of lipid bilayer enclosed particles that can be actively secreted into the extracellular milieu under physiological steady state or pathological microenvironment conditions and are widely present in all prokaryotic and eukaryotic organisms.^1^ EVs comprise several subtypes primarily distinguished by their physical size and biogenesis pathways. Larger EVs, exceeding 200 nm in diameter, are typically generated through plasma membrane shedding or budding. In contrast, small EVs (including canonical exosomes), generally under 200 nm in diameter, predominantly originate via endosomal pathway, characterized by inward budding of endosomes to form multivesicular bodies (MVBs) that subsequently fuse with the plasma membrane for extracellular release.^2^^,^^3^ However, a subset of small EVs may also form through pathways analogous to larger vesicles or directly derive from the plasma membrane.^4^ Given the current technical challenges in precisely distinguishing vesicle biogenesis mechanisms, the International Society for Extracellular Vesicles (ISEV) no longer recommends the use of traditional classification terms based on secretion pathways (e.g., exosomes, exosome-like vesicles, microvesicles, and nanovesicles).^1^

EVs play a unique dual role as a “double-edged sword” in viral-host interactions. On one hand, viruses can subvert the host cell’s EV biogenesis machinery to evade immune surveillance and achieve efficient dissemination.^5^ For instance, human immunodeficiency virus (HIV) relies on the endosomal sorting complexes required for transport (ESCRT) machinery for viral particle budding at the plasma membrane,^6^ while viruses such as rotavirus and norovirus have been demonstrated to utilize EVs as vehicles for transmission.^7^ Through this subversion, viruses efficiently package critical viral components—including viral nucleic acids, structural proteins, and even complete virions—into EVs, targeting them for specific loading. Researchers have detected hepatitis C virus (HCV) glycoproteins,^8^ Ebola virus (EBOV) nucleoproteins,^9^ and complete hepatitis B virus (HBV) virions^10^ within EVs, providing strong evidence for the role of EVs in promoting viral infection. On the other hand, EVs are also critical components of the host defense system, exerting significant antiviral functions. They can transport and present viral antigens to immune cells, effectively triggering antiviral immune responses.^11^ Additionally, EVs possess the capacity to deliver antiviral molecules to target cells. For example, following infection with herpes simplex virus type 1 (HSV-1), levels of CD9^+^ EVs enriched in the stimulator of interferon genes (STING) protein are significantly elevated, suppressing viral dissemination and enhancing host cell survival.^12^

EV biogenesis is an intricate process employing multiple mechanisms for selective cargo incorporation. Several pathways and factors regulated selective incorporation of biomolecular into exosomes, including the ESCRT machinery, the exosomal LAMP2A cargo-loading (e-LLoC) mechanism,^13^ caveolin-dependent RNA export,^14^ and ceramide-dependent microRNA (miRNA) sorting.^15^ Furthermore, microvesicle cargo selectivity is influenced by factors such as the vacuolar soluble N-ethylmaleimide-sensitive factor attachment protein receptor (v-SNARE) protein VAMP3 (vesicle-associated membrane protein 3),^16^ lipid rafts,^17^ and specific RNA-binding proteins.^18^ This EV biogenesis provided a privileged blueprint for engineering vesicles with specific cargo loading for clinical translation and the therapeutic applications.

Although virus-associated proteins within EVs released by infected cells and the hijacked biogenesis pathways represent highly promising therapeutic targets, mechanistic studies in this area remain comparatively limited. A major focus within this field is currently centered on exploiting EVs as delivery vehicles. Capitalizing on their exceptional biocompatibility and versatile cargo capacity, EVs can be engineered to efficiently encapsulate diverse therapeutic molecules, including small molecule drugs and antibodies. Systematic reviews on the role of EVs in promoting viral infection and related pathological mechanisms have been reported,^19^^,^^20^ while this article focuses on EV-mediated antiviral host defense mechanisms and the potential of EVs as antiviral therapeutics and vaccines.

## EVs trigger antiviral responses

### Initiating innate immune response

**Figure 1:**
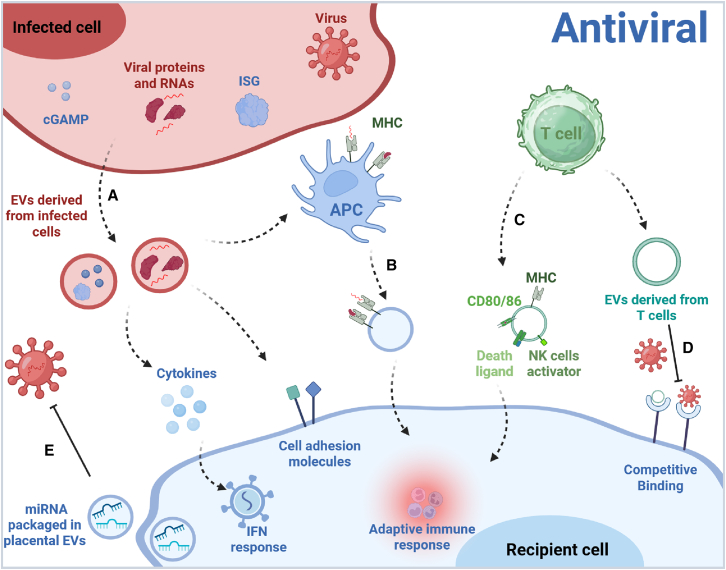
The multifunctional roles of EVs in antiviral immune responses (A) Initiating innate immune responses (top left): EVs released from virus-infected cells can carry viral components (e.g., viral RNA, proteins) acting as PAMPs. (B) Activating adaptive immune responses (center): EVs, particularly from APCs, can carry MHC molecules presenting viral peptides. (C) Delivering immunomodulatory cargo (top right): EVs serve as natural carriers for diverse immunoactive molecules and intrinsic antiviral effector molecules. Infected cells can package immune signaling molecules into EVs, transferring antiviral signals to neighboring uninfected cells. (D) Receptor competition (bottom right): EVs can competitively bind to viral particles, blocking viral attachment and entry into target host cells. (E) Direct antiviral mechanism of placental EVs (bottom left): specific miRNAs in primate placentas are packaged into EVs released by trophoblasts, providing an innate antiviral protection independent of IFN (created with BioRender.com).

In antiviral immune responses, EVs play pivotal roles, activating the immune system through multiple mechanisms (Figure 1). Primarily, EVs serve as critical mediators in initiating innate immune responses. Virus-derived components carried by EVs released from infected cells can function as pathogen-associated molecular patterns (PAMPs). These activate innate immune responses through pattern recognition receptors, such as Toll-like receptors (TLRs), in recipient cells, thereby inducing the production of antiviral cytokines such as interferons (IFNs).^21^ In HIV infection, EVs deliver viral miRNAs to macrophages, specifically activating the endosomal TLR8-mediated nuclear factor κB (NF-κB) signaling pathway, thereby stimulating tumor necrosis factor alpha (TNF-α) secretion to enhance antiviral immunity.^22^ Similarly, in late-stage influenza virus infection, apoptosis-induced degradation of host Y5 RNA (Y RNAs, typically 83–112 nucleotides long, belong to another class of small non-coding RNAs) produces a functional small RNA fragment (hsa-miR-1975), which is transported via EVs to uninfected cells, effectively initiating type I IFN-mediated antiviral defense systems.^23^

Moreover, the role of EVs in regulating innate immune responses extends beyond functioning as PAMPs^24^ as they also transfer antiviral effector molecules. Studies reveal that EVs released by infected source cells can inhibit viral replication and dissemination in neighboring cells by delivering intermediates of IFN signaling pathways or products of IFN-stimulated genes (ISGs). During HIV-1 infection, cGAMP, synthesized by cytoplasmic viral nucleic acid recognition machinery in host cells, can be loaded into EVs and co-delivered with viral particles to target cells, thereby activating the STING-dependent IFN gene pathway to exert antiviral effects.^25^ Additionally, EVs secreted by HSV-1-infected cells have been found to simultaneously contain STING protein, ISG effectors (ISG15 and ISG56), and transcripts of pro-inflammatory cytokine-encoding genes (interleukin-6 [IL-6] and IL-1β).^26^

### Activating adaptive immune response

EVs constitute a crucial bridge connecting innate and adaptive immune responses. On one hand, they induce recipient cells to secrete pro-inflammatory cytokines, thereby activating adaptive immune responses and recruiting immune cells. During dengue virus (DENV) infection, macrophage-derived EVs carrying NS3 protein and miRNA regulate vascular endothelial barrier permeability by inducing endothelial cells to secrete cytokines such as monocyte chemoattractant protein-1 (MCP-1) and IL-10, while upregulating the expression of adhesion molecules including inte**r**cellular adhesion molecule (ICAM) and VE-cadherin, thereby initiating early host defense mechanisms.^24^ EVs produced during respiratory syncytial virus infection deliver viral components to stimulate monocytes and airway epithelial cells to produce chemokines such as MCP-1/IP-10/CCL5 and CCL5/IP-10/TNF-α, respectively, for counteracting infection.^27^ In HBV infection, viral RNA delivered to macrophages via hepatocyte-derived EVs triggers the latter to secrete IFN-α-enriched EVs. These effector vesicles are internalized by neighboring hepatocytes through T cell immunoglobulin and mucin domain 1 (TIM-1) receptors, effectively inhibiting viral dissemination.^28^

On the other hand, antigen presentation represents one of the core mechanisms by which EVs participate in antiviral immune regulation. EVs released by antigen-presenting cells (APCs), such as dendritic cells (DCs), macrophages, and B cells, express major histocompatibility complex (MHC) class I or II-antigen complexes, which can directly activate CD8^+^ or CD4^+^ T cells, respectively.^29^^,^^30^^,^^31^^,^^32^ These EVs also indirectly stimulate immune responses by transferring MHC-antigen complexes to other APCs. Notably, another critical factor for EV-mediated immune enhancement lies in surface molecules (e.g., tetraspanins CD9/CD63/CD81 and integrin CD11b) that enable interactions with immune cells.^33^ In EBV-associated tumor diseases, EVs secreted by activated Vδ2-T cells are not only enriched with effector molecules such as Fas ligand, TRAIL, and NKG2D (inducing recipient cell death or activating natural killer [NK] cells) but also carry co-stimulatory ligands CD80/CD86 and MHC class I/-II complexes. These molecules, integrated into the membranes of recipient cells, significantly enhance antigen presentation efficiency in EBV-infected tumor cells for both tumor and viral antigens, thereby effectively activating CD4^+^-T-cell-mediated helper immune responses and CD8^+^-T-cell-specific cytotoxic functions.^34^

### Direct antiviral effect

In addition to modulating immune responses, EVs act as natural carriers of bioactive molecules and can directly transport antiviral-functional components. For instance, during DENV infection, infected cells release EVs containing the IFN-induced transmembrane (IFITM) protein. IFITM serves as an innate effector protein that inhibits viral entry by blocking membrane fusion between the viral envelope and host endolysosomal compartments.^35^ Similarly, EVs secreted by influenza A virus (IAV)-infected cells are also enriched with IFITM proteins. These proteins confer antiviral protection not only by restricting viral fusion and entry but also by potentiating the immune response in uninfected cells. This enhancement is mediated through the stimulated secretion of pro-inflammatory cytokines, including IL-6, TNF-α, and MCP-1.^36^ EVs isolated from HIV-infected cells have been demonstrated to carry the antiviral protein APOBEC3G,^37^ a cytidine deaminase that induces G-to-A hypermutations in viral RNA during reverse transcription after integration into viral particles, thereby suppressing viral replication.^38^ Moreover, the primate-specific C19MC miRNA cluster, exclusively expressed in the placenta during pregnancy, transfers its encoded miRNAs via trophoblastic EVs. These miRNAs suppress viral activity through a non-IFN-dependent pathway, establishing a unique innate antiviral defense mechanism.^39^^,^^40^^,^^41^

EVs can also exert antiviral effects through receptor competition.^28^ In influenza virus infection, EVs decorated on their surface with α-2,3 and α-2,6 sialic acids competitively block viral entry by binding to influenza virions prior to their interaction with target cells.^36^ Moreover, in HIV infection, CD4^+^ EVs secreted by T lymphocytes bind with high affinity to the HIV envelope glycoprotein gp120 through their surface CD4 molecules, blocking the binding of the virus to the host cell CD4 receptor by occupying the binding site required for the virus to invade the target cell.^42^

Recent research has unveiled an emerging concept—viral apoptotic mimicry—as a critical strategy employed by numerous enveloped viruses (and some non-enveloped viruses) to facilitate infection and immune evasion.^43^^,^^44^ The core mechanism involves the exploitation of the host’s conserved pathway for clearing apoptotic cells: during apoptosis, cells expose phosphatidylserine (PS) on their surface. This “eat-me” signal is recognized by widely expressed PS receptors, such as those belonging to the TIM family and the TYRO3, AXL, and MERTK (TAM) receptor family (Tyro-3, Axl, and Mer). Engagement of these receptors triggers the phagocytic removal of apoptotic cells, a process associated with immunosuppressive signaling.^45^ EVs derived from various physiological fluids (e.g., semen, saliva, and urine) exhibit potent antiviral activity at high physiological concentrations. A key common feature of these fluid-derived EVs is the enrichment of high levels of PS on their surface. They function as competitive inhibitors via the apoptotic mimicry mechanism: PS exposed on the surface of fluidic EVs preemptively occupies host cell PS receptors (e.g., Axl and TIM-1). This occupation effectively blocks the attachment and internalization of viruses that rely on the same receptor pathway for cellular entry, including Zika virus (ZIKV), DENV, West Nile virus (WNV), chikungunya virus (CHIKV), and EBOV. For instance, semen-derived EVs inhibit ZIKV infection with a half-maximal inhibitory concentration (IC_50_) of 6.02 × 10^10^ particles/mL. Crucially, this antiviral potency exhibits a direct positive correlation with the level of PS exposure. Supporting this causal link, enzymatic digestion to remove PS from the EV surface abolishes the antiviral activity, while restoration of PS restores it. This mechanism explains why bodily fluid environments deficient in PS-exposing EVs (e.g., blood EVs containing only ∼0.1 mol% PS) display significantly attenuated antiviral capacity. Furthermore, it elucidates why viruses dependent on apoptotic mimicry for entry (e.g., flaviviruses like ZIKV and DENV) primarily utilize transmission routes lacking PS-rich EVs (e.g., mosquito bites) rather than direct interpersonal contact via fluids rich in PS-exposing EVs.^46^

## EVs as therapeutic and drug delivery agents

Since the 1990s, synthetic nanoparticles (e.g., liposomes, micelles, dendrimers, polymeric, and inorganic nanoparticles) have emerged as critical tools for clinical drug delivery, improving therapeutic efficacy and reducing toxicity by enhancing the spatiotemporal distribution profiles of drugs.^47^ However, their clinical applications are constrained by limitations such as poor stability, lipid oxidation, premature drug release, and biocompatibility issues caused by exogenous components, which may induce allergic reactions and immunogenicity risks.^48^^,^^49^ In contrast, EVs possess a natural lipid bilayer membrane structure that enables effective encapsulation of biomolecules such as nucleic acids and proteins while resisting enzymatic degradation.^50^^,^^51^ Furthermore, EVs exhibit high homology with host cells, allowing them to evade immune clearance and traverse physiological barriers (e.g., the blood-brain barrier and placental barrier) for targeted delivery.^52^^,^^53^ These biological characteristics confer superior safety, targeting efficiency, and drug protection profiles to EVs compared to traditional synthetic carriers, highlighting their enhanced clinical potential. Table 1 compares EVs and synthetic nanoparticles.

**Table 1:** Comparison between EVs and synthetic nanoparticles

	EVs	Synthetic nanoparticles
Biocompatibility	natural carrier, low immune clearance	may cause an immune response
Targeting capability	natural targeting is excellent, but heterogeneity is high	programmable targeting
Intracellular delivery	efficient	relying on endocytosis, an escape mechanism needs to be designed
Drug loading and preparation	complex process and difficult to scale up	technology is mature and easy to scale
Immunogenicity	natural immune stimulation and may carry inhibitory molecules	adjuvant effect is strong, high controllability
Clinical translation	early experiments were safe, but efficacy still needs to be validated	practical application

Currently, EV drug loading methods primarily involve passive and active approaches.^54^ Passive loading (e.g., co-incubation) relies on concentration gradients to facilitate spontaneous diffusion across EV membranes. While operationally simple, this method suffers from low loading efficiency and limited molecular selectivity. In contrast, active loading employs techniques such as electroporation, ultrasonication, or chemical permeabilization to disrupt membrane integrity, enhancing drug incorporation. Although this increases drug payload, the physicochemical stress may compromise EV membrane integrity, which may impair their intrinsic delivery capabilities. Table 2 summarizes the comparative advantages and disadvantages of these EV loading strategies.

**Table 2:** EV loading strategies

Method	Advantages	Disadvantages	Common drug species
Cell co-culture	convenient, mild	low loading efficiency	small molecule drugs, synthetic oligonucleotides
Sonication	high loading efficiency	heat generation, compromise membrane integrity, destruction of active ingredients	siRNAs, proteins, small molecule drugs
Electroporation	high loading efficiency, controllable	aggregation, additional equipment	small DNAs, microRNAs, siRNAs
Freeze-thaw cycle	convenient, mild, suitable for small and medium molecule drug loading	aggregation, change the size of EVs	protein, small molecule drugs
Extrusion	high loading efficiency, uniform particle size	compromise membrane integrity	protein, small molecule drugs
Liposome fusion with EV	high loading efficiency, mild	difficult purification	small molecule drugs, microRNAs, siRNAs

### HIV

Engineered EVs, leveraging their precise targeting capabilities and low immunogenicity, have emerged as a pivotal platform for delivering antiviral therapeutics, demonstrating significant promise in overcoming critical bottlenecks in HIV treatment. To activate the latent HIV-1 reservoir, researchers achieved targeted encapsulation of the labile viral transcription activator Tat protein into the EV lumen, while simultaneously displaying the CD4-targeting domain derived from IL-16 on the vesicle surface. This engineering strategy enabled specific delivery to CD4^+^ T cells. The resulting engineered EVs efficiently stimulated transcription from latently infected proviruses, inducing a reactivated state susceptible to clearance by antiretroviral drugs, while avoiding significant aberrant immune responses.^55^ Complementary strategies focused on enhancing targeted cell clearance involve decorating the EV surface with high-affinity HIV-1-specific antibodies (e.g., single-chain variable fragment [scFv] derived from the 10E8 monoclonal antibody) and concurrently packaging therapeutic payloads (such as curcumin or the pro-apoptotic miRNA-143) within the vesicle lumen. This approach creates a dual-functional delivery system capable of both precise recognition of infected cells and induction of apoptosis. Experimental evidence confirms that these antibody-targeted, drug-loaded EVs specifically recognize and kill cells expressing HIV envelope proteins (including within brain reservoir sites), with efficacy validated in animal models.^56^ Furthermore, strategies employing EVs to deliver gene editing or epigenetic regulatory tools for achieving durable viral suppression have been developed. For instance, zinc finger protein ZFP-362 delivered via EVs can bind specifically to the HIV-1 promoter sequence. By recruiting the catalytic domain of DNA methyltransferase 3A (DNMT3A), it induces stable, long-term methylation modifications at the promoter region, resulting in effective epigenetic silencing of the integrated provirus. This exosomal delivery of ZFP-362 effectively suppressed HIV replication across multiple tissues, including bone marrow, spleen, and brain, in humanized NSG mouse models.^57^ Collectively, these studies demonstrate the robust capabilities and versatile application potential of engineered EVs for the targeted delivery of diverse effector molecules to achieve HIV reservoir activation, clearance of infected cells, or long-term suppression.

### SARS-CoV-2

EVs, particularly mesenchymal-stem-cell-derived EVs (MSC-EVs), demonstrate substantial promise for developing therapeutic strategies against COVID-19. The immunomodulatory properties of MSCs involve modulating effector functions in immune cells. Published studies indicate MSCs suppress pulmonary infiltration and resolve pulmonary edema. These cells possess multipotency, regenerative capacity, and self-renewal capabilities, enabling them to suppress immune responses and differentiate into type II alveolar epithelial cells *in vitro*.^58^ In a phase II clinical trial (NCT03608592) evaluating patients with acute lung injury (ALI) and acute respiratory distress syndrome (ARDS), MSCs demonstrated significant anti-inflammatory activity.^59^ Findings from safety trials suggest MSCs can ameliorate SARS-CoV-2-associated cytokine storm (CS) and ARDS, potentially offering a prospective therapeutic approach for chronic respiratory dysfunction and pulmonary fibrosis. Furthermore, MSC-EVs administered via inhalation to COVID-19 patients in a separate investigation (NCT04276987) proved safe and well tolerated, with no predefined adverse events reported. This therapy significantly improved lymphocyte counts, reduced inflammatory markers including IL-6, and promoted pulmonary repair while mitigating fibrosis in critically ill COVID-19 patients.^60^ A recent prospective non-randomized open-label cohort study evaluated the efficacy of allogeneic bone marrow MSC-derived EVs (BM-MSC-EVs) in 24 SARS-CoV-2-infected subjects with moderate-to-severe ARDS. Results confirmed an excellent safety profile (no treatment-related adverse events) alongside significantly improved oxygen saturation. This improvement correlated with decreased absolute neutrophil counts, increased lymphocyte counts, and reduced levels of acute-phase reactants such as C-reactive protein, ferritin, and D-dimer.^61^ Additionally, EVs derived from specific immune cell types or engineered to carry antiviral components show direct therapeutic potential against COVID-19. For instance, an ongoing clinical trial (NCT04389385) proposes using EVs derived from SARS-CoV-2-specific fragment-peptide-activated T cells to treat early-stage COVID-19. These EVs likely harbor potent mediators, including IFN-gamma (IFN-γ), which may effectively antagonize SARS-CoV-2 infection by directly interacting with the virus or protecting susceptible cells from viral recognition.^62^^,^^63^ Since recombinant soluble angiotensin-converting enzyme 2 (rsACE2) protein can competitively inhibit SARS-CoV-2 binding to ACE2-expressing cells *in vitro* and block infection,^64^ EVs harboring ACE2 represent a potential therapeutic strategy to limit viral infection progression *in vivo*.^62^^,^^63^ Supporting this, studies have observed ACE2-containing EVs binding to SARS-CoV-2 via the viral spike (S) protein, indicating engineered EVs are a promising strategy for blocking SARS-CoV-2 infection.^65^ Further evidence demonstrates that MSC-EVs decorated with the S protein occupy ACE2 receptors on type II alveolar epithelial cells. This competitive binding effectively blocks cellular uptake of SARS-CoV-2 and protects cells from viral infection.^66^ Through rigorous and comprehensive research, engineered EVs hold significant promise for playing a vital role in combating COVID-19 and other infectious diseases.

### Hepatitis virus

The integration of gene-editing technologies with EVs has further broadened the frontiers of antiviral therapy.^67^^,^^68^ For instance, key components of the CRISPR/Cas9 system—including guide RNA (gRNA) and Cas9 protein, renowned for their precision in genome editing—have been successfully packaged into EVs and delivered functionally. Studies confirm that endogenously derived EVs can successfully deliver functional Cas9 protein paired with HBV-specific gRNA, facilitating the efficient cleavage of HBV DNA in hepatocyte models (e.g., Huh7 cells). This demonstrates the feasibility of applying EV-mediated delivery for gene therapy targeting viral diseases.^69^ Concomitantly, EVs originating from diverse cell types exhibit unique therapeutic value. EVs derived from specific tumor cells or DCs pulsed with such EVs can induce potent anti-tumor immunity and remodel the tumor immune microenvironment through direct or indirect immunomodulatory mechanisms, such as reducing the accumulation of regulatory T cells (Tregs) within the tumor milieu.^70^ Conversely, EVs secreted by umbilical cord mesenchymal stem cells (uMSC-EVs) have been demonstrated to effectively inhibit HCV infection and replication *in vitro* with low cytotoxicity.^71^ Furthermore, dendritic-cell-derived EVs enriched with tumor-associated antigens (TAAs) show promise as innovative cell-free immunotherapeutic vaccines. For example, in hepatocellular carcinoma (HCC) models, dendritic-cell-derived EVs enriched with alpha-fetoprotein (AFP) elicited robust antigen-specific immune responses, consequently delaying tumor progression and significantly extending survival.^72^

### Other viruses

In ZIKV infection—a pathogen known to cause fetal neurological developmental defects—the absence of effective preventive vaccines, combined with the necessity to traverse the placental barrier and meet stringent safety requirements for fetal therapy, poses a major global public health challenge in treating such conditions.^73^ To address this, researchers innovatively developed engineered EVs loaded with IFITM3, an intrinsic antiviral protein localized to endosomal-lysosomal membranes that inhibits viral replication by blocking membrane fusion between viral envelopes and host cells and is highly expressed in the placenta. Animal studies confirmed that these engineered EVs not only fully preserved IFITM3 bioactivity but also achieved targeted delivery to placental tissue due to natural tropism, markedly reducing fetal viral infection rates.^74^

In the treatment of HSV-1, recent research has innovatively exploited the high-affinity interaction between the human immunoglobulin G (IgG) Fc fragment and *Staphylococcus aureus* protein A (SpA). Specifically, the human Fc domain was anchored to the EV transmembrane scaffold protein prostaglandin F2 receptor negative regulator with deleted C-terminal region (PTGFRN-Δ687), while the SpA domain was fused to the Cas9 protein. This engineering strategy significantly enhanced (nearly 2-fold) the enrichment efficiency of Cas9 ribonucleoproteins (RNPs) within EVs. Subsequent surface modification with the rabies virus glycoprotein-derived RVG29 peptide endowed the EVs with targeted neurotropic delivery capability, enabling precise transport to HSV-1 latent sites in neural tissues, such as the trigeminal ganglia. Concurrently, co-incorporated vesicular stomatitis virus glycoprotein (VSV-G) mediated efficient endosomal/lysosomal escape, facilitating the release of the Cas9/sgUL29 complex. This complex specifically cleaves the essential viral gene UL29. Experimental validation in murine models demonstrated that EVs engineered via this multifaceted approach achieved a 90% significant reduction in viral load within the trigeminal ganglia. Crucially, animal survival rates increased from 0% to 80%, while exhibiting a remarkably low off-target rate (<1%), demonstrating a unified profile of high therapeutic efficacy and safety. This strategy represents a novel and promising direction for targeted antiviral therapy.^75^

The precision-targeting attributes of EVs are further harnessed in highly active antiretroviral therapy (HAART). For HIV infection, although the viral transactivator protein Tat effectively activates latent HIV-1 provirus, its free form is vulnerable to proteolytic degradation and difficult to target infected cells specifically. To overcome this limitation, researchers directionally encapsulated HIV-1 Tat protein within the intravesicular membrane of EVs and fused a CD4-targeting domain derived from IL-16 onto the surface. These engineered EVs achieve precise delivery to CD4^+^ T cells, efficiently activating transcription of latent virus and inducing a drug-susceptible active state amenable to clearance by antiretroviral agents, while avoiding aberrant immune reactions.^55^ Additional research developed a therapeutic strategy with dual functions of precise targeting recognition and synergistic induction of apoptosis by functionalizing the EV surface with high-affinity HIV-1-specific antibodies while encapsulating therapeutic molecules such as curcumin or pro-apoptotic miRNA-143 within the vesicle.^56^

The integration of gene editing technologies with EVs further expands the potential for antiviral therapy.^67^^,^^68^ Taking HSV-1 treatment as an example, recent research has innovatively leveraged the high-affinity interaction mechanism between the Fc domain and SpA. Specifically, the human Fc domain was anchored to the EV membrane protein PTGFRN-Δ687, while the SpA domain was conjugated to the Cas9 protein. This strategy significantly enhanced the enrichment efficiency of Cas9 RNPs within EVs (by nearly 2-fold). The EVs were further modified with RVG29 peptide to confer neural targeting capability, enabling their precise delivery to neuronal tissues harboring latent HSV-1, such as the trigeminal ganglia. Coupled with VSV-G-protein-mediated lysosomal escape, this facilitated the release of the Cas9/sgUL29 complex to target and cleave the critical viral gene UL29. In mouse models, this EV engineering strategy yielded a 90% reduction in trigeminal ganglia viral load, elevated survival from 0% to 80%, and limited off-target effects to <1%, effectively reconciling high antiviral efficacy with safety to define a novel antiviral therapeutic approach.^75^

## EV-based vaccines

EVs demonstrate important value in vaccine development, particularly in the antiviral field, due to their unique capacity for antigen delivery and presentation. This application concept originated from the observation that EVs released from B lymphocyte cell lines can specifically carry peptide/MHC class II complexes and effectively activate antigen-specific immune responses.^29^ As naturally derived nanoscale carriers, EVs not only efficiently deliver immunogenic viral antigenic epitopes but also directly activate immune cell recognition mechanisms through their surface presentation of immunostimulatory signals. Current antiviral vaccines are primarily based on modified live or attenuated viruses, inactivated viruses, DNA and RNA vector vaccines, viral subunits, or single peptides. However, some developed vaccines confer limited protective immunity, fail to elicit durable protection, and carry the risk of reversion to virulence. These limitations underscore the need for alternative strategies in vaccine design.^76^

EV-based vaccines (Table 3) offer distinct advantages over conventional vaccines in delivery strategy, immune activation, safety profile, and manufacturing cost. Regarding delivery, EVs leverage their native lipid bilayer membrane and surface proteins to efficiently target antigens to immune cells like DCs while protecting bioactive cargo from degradation, whereas conventional vaccines, such as mRNA formulations, rely on synthetic lipid nanoparticles (LNPs), which exhibit lower stability and delivery efficiency. In immune activation, EV vaccines can co-deliver multiple native pathogen-associated membrane proteins, mimicking natural infection to elicit multi-pathway immune responses, robust T cell immunity, and durable immune memory; in contrast, traditional inactivated or subunit vaccines primarily induce humoral immunity, often requiring adjuvants to enhance immunogenicity, and generally elicit weaker T cell responses and shorter-lasting protection. From a safety perspective, EVs possess inherent biocompatibility, avoiding risks associated with conventional platforms such as virulence reversion in live-attenuated strains, local inflammation from aluminum adjuvants, or systemic adverse effects linked to LNP carriers. Cost-wise, although purification costs for animal-derived EVs remain higher, plant-derived EVs offer substantial potential for cost-effective industrial-scale production, while the complex synthesis and stringent cold-chain requirements for nucleic acid vaccines drive their overall cost upward.^77^^,^^78^^,^^79^

**Table 3:** EV-based antiviral vaccines

Virus	Vaccine	Antigen	Route of administration	EV source	Mechanism & characteristics	References
HIV	Gp120-Texo	HIV Gp120	intravenous injection	CD8^+^ T cells uptaking DC-EV	overcomes immune limitations of DC dysfunction and CD4^+^ T cell deficiency; induces HIV gp120-specific CTL responses; exhibits therapeutic anti-tumor effects and long-term immune memory	Nanjundappa et al.^80^; Nanjundappa et al.^81^
	Gag-Texo	HIV Gag	intravenous injection	CD8^+^ T cells uptaking DC-EV	Gag possesses richer conserved epitopes; it effectively reverses T cell dysfunction and rebuilds anti-viral immunity in chronic infection models	Rong et al.^82^; Wang et al.^83^
SARS-CoV-2	lung-EVs mRNA vaccine	SARS-CoV-2 Spike	inhalation	lung-spheroid-cell-derived EVs	native lung tissue tropism for targeted delivery; lyophilized into room temperature stable dry powder (≥28 days); induces strong mucosal immunity: significantly higher specific IgG and sIgA levels in BALF than conventional lipid carriers, enabling rapid respiratory virus clearance	Popowski et al.^84^
	milk-EVs mRNA vaccine	SARS-CoV-2 RBD	take orally	bovine milk-derived EVs	purified via density gradient ultracentrifugation; DOTAP mediates mRNA loading; overcomes gastrointestinal barrier; induces sustained neutralizing antibody responses; low-cost, easy production	Zhang et al.^85^
	HEK293F-EV dual-antigen mRNA vaccine	SARS-CoV-2 spike & nucleocapsid fusion protein (LSNME) mRNA	intramuscular injection	HEK293F cell-derived EVs	cationic liposomes encapsulating mRNA co-incubated with EVs (high encapsulation efficiency >90%); induces sustained antibody responses (anti-S/anti-N) and T cell immunity (CD4^+^/CD8^+^ Th1 polarization); no significant toxicity	Tsai et al.^86^
	RBD-OMV intranasal vaccine	SARS-CoV-2 RBD	intranasal immunization	OMV of Salmonella	SpyTag/SpyCatcher system for covalent RBD conjugation to OMV surface; induces dual immunity: high-titer cross-neutralizing antibodies (systemic) and anti-RBD IgG in BALF (mucosal)	Jiang et al.^87^
HBV	DC-EV vaccine	HBV peptide, MHC I/CD40/CD86/IL-6	intravenous injection	DC-derived EV	spleen targeting: enhances CD8^+^ T cell proliferation and CTL function; liver targeting: promotes M2→M1 macrophage conversion; combined with Alum-adjuvanted vaccine, induces comparable HBV-specific IgG levels and synergistically enhances CD4^+^ T cell activation & central memory T cell proportion	Hu et al.^88^
	EV adjuvant		subcutaneous injection	THP-1-cell-derived EVs	subcutaneous immunization after physical mixing with HBsAg; accelerates early anti-HBsAg IgG production; significantly increases cytokine secretion (e.g., IFN-γ), inducing robust Th1-type cellular immune responses	Jesus et al.^89^
RSV	SyBV vaccine	RSV preF-ClyA fusion protein	intramuscular injection	synthetic bacterial vesicles	uniform size, high protein yield, enhanced storage stability, and biosafety; retains ability to activate TLR4/MyD88/NF-κB signaling; promotes DC maturation; induces high-titer preF-specific IgG, Th1 polarization, and CTL activation	Meng et al.^90^
Multiepitope vaccine	Nefmut EV platform	multi-viral antigens (EboV VP24/VP40/NP, Flu NP, CCHFV NP, WNV NS3, HCV NS3)	intramuscular injection	muscle-cell-derived EVs loaded with Nefmut	induces significant antigen-specific CD8^+^ T cell responses and cytotoxic activity; compatible with diverse viral antigens (structural/non-structural) across a range of molecular weights	Anticoli et al.^91^
	StealthX EV platform	Influenza H3 HA, RSV F protein, SARS-CoV-2 Delta spike	intramuscular injection	genetically engineered HEK-293F-derived EVs	monovalent vaccine (low dose) induces strong humoral and cellular immunity without adjuvant; trivalent formulation induces high-level neutralizing antibodies and cellular immunity against all three pathogens simultaneously, with no immune interference	Cacciottolo et al.^92^

### HIV

Acquired immunodeficiency syndrome (AIDS), caused by HIV, is a lethal disorder characterized by viral targeting of CD4^+^ T lymphocytes, leading to progressive immune collapse. While highly active antiretroviral therapy (HAART) effectively suppresses viral replication, it presents issues such as drug toxicity and drug resistance, and some patients experience poor immune reconstitution after treatment.^93^ Consequently, there is a critical imperative for novel immunotherapeutic strategies. Vaccine designs based on EVs represent a significant research avenue. The work of Nanjundappa et al. demonstrated the development of a Gp120-Texo vaccine through the delivery of gp120-specific EVs, released by DCs transfected with the pcDNAgp120 plasmid, to concanavalin A (ConA)-activated CD8^+^ T cells from C57BL/6 mice. Crucially, this vaccine directly elicits HIV gp120-specific CD8^+^ cytotoxic T lymphocyte (CTL) responses *in vivo*, bypassing conventional requirements for host CD4^+^ T cell help and DC-mediated antigen presentation. Furthermore, it induced durable protective immunity against gp120-expressing B16 melanoma tumors in mice, providing a new pathway for AIDS patients with DC dysfunction and CD4 T cell deletion.^80^

To validate this strategy in a model simulating human immunity, subsequent research utilized recombinant DNA technology to create an adenoviral vector expressing HIV-1 gp120 (AdVgp120). This vector transfected bone-marrow-derived DCs (BMDCs) from HLA-A2 transgenic mice, generating humanized gp120-specific EVs. These EVs were then loaded onto ConA-activated CD8^+^ T cells, also derived from HLA-A2 transgenic mice, to construct a humanized Gp120-Texo vaccine. Experiments confirmed that within the HLA-A2 transgenic mouse model, this humanized vaccine efficiently stimulates a gp120-specific CTL response independent of CD4^+^ T cells. Critically, it also induced significant therapeutic antitumor effects and promoted long-term immunological memory.^81^

Given that the HIV Gag antigen has more conserved epitopes and greater immunogenicity advantages compared to Gp120, Wang et al. further developed a Texo vaccine variant based on the Gag antigen (Gag-Texo). This was achieved by transfecting DCs with the adenoviral vector AdVGag to generate Gag-specific EVs, which were subsequently loaded onto ConA-activated CD8^+^ T cells to form the vaccine. In both wild-type C57BL/6 and transgenic HLA-A2 mice, the Gag-Texo vaccine successfully stimulated Gag-specific CTL responses, inducing protective and durable immunity against Gag-expressing B16 melanoma.^82^ In the subsequently established AdVova-adenovirus-induced chronic infection model (which exhibits high expression of PD-1/LAG-3 inhibitory receptors in CTLs, functional exhaustion, and unresponsiveness of initial CD8^+^ T cells), it was confirmed that the Gag-Texo vaccine could induce therapeutic immunity against Gag-expressing tumors during chronic infection, significantly reducing the burden of established pulmonary metastases.^83^ Collectively, these findings demonstrate that the strategy based on Texo vaccines, particularly the use of variants of conserved antigens like Gag, has unique therapeutic advantages and broad clinical application prospects in reversing T cell dysfunction, reconstructing effective immunity to combat chronic infections including HIV.

### SARS-CoV-2

In the global COVID-19 pandemic response, EV-based vaccines for SARS-CoV-2 have been extensively studied. Scientists engineered novel vaccine carriers with targeted delivery capabilities and enhanced stability by modifying EVs from diverse sources. Popowski et al. developed a novel inhalable mRNA vaccine based on the natural pulmonary tissue affinity of EVs (lung-EVs) secreted by human lung spheroid cells (LSCs).^84^ This research loaded mRNA encoding the SARS-CoV-2 spike protein into lung-EVs and employed freeze-drying technology to create a room-temperature-stable dry powder formulation (shelf life ≥28 days), circumventing the cold-chain dependence of conventional mRNA vaccines.^94^ Animal studies demonstrated significantly stronger mucosal immune responses than traditional lipid carriers, with markedly elevated SARS-CoV-2-specific IgG and secretory IgA (sIgA) levels in bronchoalveolar lavage fluid. Challenge experiments further confirmed its ability to rapidly clear respiratory viruses, underscoring the breakthrough potential of organ-derived EVs in optimizing delivery efficiency and enabling needle-free administration.^84^

Zhang et al. reported an orally administered SARS-CoV-2 RBD mRNA vaccine based on bovine-milk-derived EVs, which successfully induced sustained neutralizing antibody responses in mice via duodenal delivery. The study innovatively employed density-gradient-ultracentrifugation (UC)-purified bovine milk EVs as natural delivery vehicles. Utilizing a cationic-liposome-mediated loading strategy, RBD-encoding mRNA was efficiently encapsulated, overcoming the gastrointestinal barrier limitations inherent to oral vaccines. Experiments confirmed that this vaccine facilitated high-efficiency RBD protein expression in *in vitro* cell models and elicited specific neutralizing antibodies in mice, demonstrating its potential to establish an immune barrier against SARS-CoV-2 through the oral route. This delivery system, based on food-grade biomaterials, offers the combined advantages of low cost and facile production, thereby pioneering a novel approach for vaccine development.^85^

Tsai et al. implemented a similar strategy to engineer EVs for vaccination purposes. They developed a novel vaccine by loading mRNA encoding the SARS-CoV-2 spike protein and a nucleocapsid-membrane fusion protein (LSNME, a chimera containing spike/membrane/envelope protein fragments fused to human Lamp1 protein) into EVs derived from HEK293F cells. The study employed a strategy involving cationic-lipid-mediated mRNA followed by co-incubation with EVs, achieving high-efficiency encapsulation (>90%). In the C57BL/6J mouse model, intramuscular injection of dual-antigen mRNA-loaded EVs induced sustained, dose-dependent anti-spike and anti-nucleocapsid antibody responses lasting up to 3 months, alongside significant activation of antigen-specific CD4^+^ and CD8^+^ T cell proliferation. Notably, spike-protein-specific T cells exhibited a classic Th1-polarized phenotype, characterized by a significant increase in IFN-γ production without significant changes in IL-4. Repeat dosing experiments confirmed sustained target protein expression by the platform for at least 10 weeks. Furthermore, histopathological analysis revealed no signs of injection site or systemic toxicity, highlighting its potential as a safe and efficient mRNA delivery system.^86^

Jiang et al. reported an innovative intranasal vaccine strategy based on bacterial outer membrane vesicles (OMVs). The study utilized genetically engineered, attenuated *Salmonella typhimurium* to covalently decorate OMVs with the recombinant SARS-CoV-2 RBD, expressed in mammalian cells, via the SpyTag/SpyCatcher system, forming RBD-OMV complexes. Intranasal immunization with RBD-OMV in Syrian golden hamster models elicited a dual immune response: a systemic immune response characterized by high-titer cross-neutralizing antibodies (against wild-type and Delta variant strains) and a mucosal immune response evidenced by significantly elevated anti-RBD IgG in bronchoalveolar lavage fluid. Challenge experiments confirmed that immunized animals were completely protected from weight loss, exhibited 2–3 orders of magnitude reduction in viral load in lung tissue and respiratory tract secretions, and showed significantly alleviated pulmonary pathological damage. This study highlights the potential of the OMV for developing next-generation vaccines, offering advantages such as an efficient antigen delivery mechanism mimicking natural pathogens, the convenience of intranasal administration, inherent adjuvant activity (derived from OMV components), and flexibility in addressing variant strains.^87^

### HBV

HBV infection represents a major global public health concern, capable of leading to severe consequences including chronic hepatitis, cirrhosis, and hepatocellular carcinoma. Building on recent research by Hu et al., APC-derived EVs demonstrate an innovative dual-pathway strategy for HBV therapy. By exposing APCs, such as DCs, to HBV-specific peptides and the immune activator lipopolysaccharide (LPS), the production of EVs carrying immunogenic molecules (significantly elevated MHC class I and CD40/CD86) and inflammatory cytokines (IL-6 and TNF-α) was induced. Following intravenous injection, these EVs exhibited precise tropism to the spleen and liver via homing receptors (e.g., CCR7). Spleen-targeting EVs significantly enhanced CD8^+^ T cell proliferation and cytotoxic function (enhanced specific killing *in vivo*, upregulation of FasL expression, and increase in granzyme B secretion) through direct antigen presentation and APC activation. Conversely, liver-targeting EVs reprogrammed the local immune microenvironment by promoting the conversion of M2 macrophages toward a pro-inflammatory M1 phenotype while simultaneously reducing the proportion of regulatory T cells (Tregs). When combined with Alum-adjuvanted, this strategy not only induced HBV-specific IgG antibody levels comparable to Alum-adjuvanted alone but also synergistically enhanced CD4^+^ T cell activation and boosted the proportion of central memory T cells. This dual potentiation of both humoral and cellular immunity provides a novel approach for the treatment of chronic hepatitis B.^88^

Furthermore, Jesus et al. conducted the first systematic investigation into the use of unmodified EVs as adjuvants for recombinant hepatitis B surface antigen (HBsAg) vaccine. The study involved isolating EVs from (LPS-stimulated human monocytic cells (THP-1) and physically co-administering them with HBsAg via subcutaneous immunization in C57BL/6 mice. Experimental results revealed that EVs themselves possess intrinsic immunostimulatory activity, capable of inducing splenocytes to secrete pro-inflammatory cytokines (e.g., TNF-α, RANTES, and IL-1β). When combined with HBsAg, while the level of humoral immune response was comparable to that elicited by the antigen alone, co-administration significantly accelerated early anti-HBsAg IgG production (with partial seroconversion observed as early as 14 days post-primary immunization). Crucially, co-administration significantly boosted the secretion of cytokines such as IFN-γ and induced a potent Th1-polarized cellular immune response. This study demonstrated for the first time that EVs, even without antigen modification and merely via simple physical co-administration, can function as effective adjuvants for HBV vaccines. Their core value lies in enhancing Th1-polarized cellular immunity and accelerating early antibody responses, offering a novel approach for developing new-generation hepatitis B vaccines.^89^

### RSV

Meng et al. developed synthetic bacterial vesicles (SyBV) technology, offering a transformative advance over traditional OMVs for respiratory syncytial virus (RSV) vaccines. While OMVs derived from Gram-negative bacteria (e.g., *Escherichia coli*) represent attractive vaccine carriers due to their natural nanostructure and inherent immunostimulatory properties, they face limitations including compositional heterogeneity, complex large-scale manufacturing processes, and potential proinflammatory risks.^95^ Meng et al.’s SyBV, engineered using bacterial membrane engineering techniques, effectively overcomes these drawbacks: a dedicated lysis-purification process removed cytoplasmic contaminants, while homogenization processing enabled size stabilization (85 nm, PDI<0.07), achieving a 6-fold increase in protein yield and ensuring long-term storage stability at 4°C. SyBVs retain the ability to activate TLR4/MyD88/NF-κB signaling pathways but induce significantly reduced secretion of proinflammatory cytokines (TNF-α and IL-6), demonstrating superior biosafety profiles compared to native OMVs. Within the context of an RSV vaccine, intramuscularly administered SyBV-preF complexes exhibited targeted accumulation within inguinal lymph nodes, promoting DC maturation and upregulating co-stimulatory molecules CD80/CD86. Animal studies confirmed that immunization with SyBV-preF induced preF-specific IgG antibody titers 10-fold higher than an Alum-adjuvanted control group. Furthermore, it significantly enhanced Th1-polarized immune responses (as evidenced by a 3.2-fold increase in IFN-γ secretion) and cytotoxic T cell responses, while maintaining superb biocompatibility.^90^

### Multiepitope vaccines

To broaden the applicability of EVs as vaccine carriers against diverse viral pathogens, Anticoli et al. developed an innovative and versatile platform based on the genetically modified HIV-1 Nef protein.^91^ This platform employs genetic engineering to convert the wild-type Nef protein into a loss-of-function mutant (Nefmut), effectively circumventing its deleterious immunomodulatory functions, including CD4 receptor downregulation, enhanced viral infectivity, and inhibition of MHC class I molecule expression. Capitalizing on the unique property of Nefmut for efficient and specific enrichment within EVs, the team constructed a cytomegalovirus (CMV)-promoter-driven DNA vector. This vector enables the expression of various viral antigens as C-terminal fusion partners with Nefmut. Experiments demonstrated that heterologous antigens from diverse viruses—such as EboV VP24, VP40, and NP; influenza A virus (Flu) NP; Crimean-Congo hemorrhagic fever virus (CCHFV) NP; West Nile virus (WNV) NS3; and HCV NS3—all formed stable fusion proteins with Nefmut. Following *in vivo* expression, these fusion proteins were efficiently anchored within released EVs. Murine model studies revealed that intramuscular injection of the DNA vectors encoding the distinct fusion proteins successfully elicited robust antigen-specific CD8^+^ T cell responses in all tested groups. Crucially, isolated antigen-specific CD8^+^ T cells exhibited significant cytotoxic activity against target cells pulsed with specific cognate peptides or stably expressing the corresponding target antigens. This cytotoxic activity was demonstrably dose-dependent, increasing with the effector-to-target cell ratio. This study established the broad applicability of this platform for inducing functional CTL immunity, effectively accommodating viral antigens of diverse origins (structural/non-structural proteins) and varying molecular weights.^91^

In another research, Cacciottolo et al. utilized the StealthX EV vaccine platform to genetically engineer the fusion of coding sequences for influenza virus H3 hemagglutinin (HA), respiratory syncytial virus (RSV) fusion (F) protein, and SARS-CoV-2 Delta spike (S) protein to the extracellular domain of the tetraspanin protein CD9. Exploiting the CD9 transmembrane domain, this strategy directed the antigens for targeted anchoring onto the exosomal membrane surface, successfully generating three engineered EVs (STX-H3, STX-RSV, and STX-S). Intramuscular immunization of mice with monovalent EV vaccines (STX-H3: 10 ng, STX-RSV: 132 ng, and STX-S: 10 ng) without adjuvant elicited potent humoral immunity (anti-H3 IgG titers increased by 4,500-fold versus control) and cellular immunity (2- to 4-fold increase in IFN-γ-secreting cells post-antigen stimulation). A key breakthrough was achieved using a “mix and match” strategy for the trivalent vaccine (STX-H3 + STX-RSV + STX-S). Simultaneous delivery of the three antigens induced high titers of neutralizing antibodies against influenza, RSV, and SARS-CoV-2 in all immunized animals. Critically, these neutralizing antibody titers were comparable to those elicited by the corresponding monovalent vaccines. Furthermore, the number of IFN-γ-secreting cells specific for each individual antigen within the trivalent group was equivalent to those observed in the monovalent immunization groups, demonstrating that the exosomal carrier effectively circumvents antigenic interference. The core advantage of this platform resides in its modular design: By independently preparing single-antigen EVs and enabling their flexible combination, it facilitates single-injection activation of multi-pathogen immune responses and achieves this with nanogram-range antigen dosing (representing 2–3 orders of magnitude reduction compared to conventional vaccines). This provides a scalable technological pathway for rapidly responding to viral variants and emerging outbreaks, holding significant potential to transform the landscape of multivalent viral vaccine development.^92^

## Challenges and conclusion

The separation step remains a primary bottleneck in the scalable production of EVs. Ultracentrifugation (UC), the classical technique for EV isolation, effectively separates EVs based on differences in size and density and is amenable to large-scale sample processing.^96^ However, UC is time-consuming, requires specialized equipment, and is susceptible to lipoprotein contamination, and its high centrifugal forces can compromise vesicle functionality.^97^ Combining UC with density gradient centrifugation can mitigate damage risks and enhance purity, but at the expense of significantly lower yield and increased operational complexity.^98^ Precipitation methods, such as polyethylene glycol (PEG)-based precipitation, offer straightforward and efficient separation by exploiting changes in solubility or aggregation, making them suitable for large-scale isolation. Nevertheless, precipitation can interfere with EV structure and function and often results in co-precipitation of impurities.^99^^,^^100^ Tangential flow filtration (TFF) achieves separation by leveraging tangential fluid flow and membrane pore size exclusion. Its short processing time, scalability, and capability for continuous operation establish it as a standard purification method for liposome production.^101^^,^^102^ Given the structural similarities between EVs and liposomes and TFF’s relatively low shear stress, which is gentler on vesicle integrity compared to UC, TFF is also considered an optimal solution for large-scale production of EVs.^103^ Meanwhile, it suffers from inadequate purity, as substantial quantities of co-purified protein and lipid impurities necessitate additional purification steps, increasing time requirements and reducing overall yield.^104^^,^^105^ To enhance purity, the combination of TFF with size exclusion chromatography (SEC) has been employed; this strategy efficiently removes various protein contaminants, achieves yields comparable to UC, and preserves EV bioactivity, size distribution, morphology, and protein composition.^106^ In recent years, affinity chromatography (AC), relying on antigen-antibody interactions, has enabled rapid isolation of EVs with high purity. Yet, its high cost, dependency on specific reagents, and challenges in scaling significantly limit its widespread application.^107^ Given the limitations of existing separation methods and their inability to serve as a “gold standard,” it is crucial to develop personalized separation and purification strategies based on actual needs and application orientation. For future large-scale applications, there is an urgent need to develop novel EV separation technologies that achieve high yield and high purity while ensuring structural integrity and maintaining biological activity. At the same time, these technologies must also meet the comprehensive requirements of large-scale production, including scalability, cost-effectiveness, and high-throughput processing capabilities.

EVs are inherently heterogeneous populations, exhibiting significant molecular and compositional variations even among individual vesicles originating from the same source.^2^ This heterogeneity encompasses not only physical properties such as size,^108^ density,^109^ or viscoelastic differences^110^ but also molecular composition, including the non-uniform distribution of membrane proteins.^111^ In contrast to synthetic drug delivery carriers, whose physicochemical properties are typically stringently controlled within narrow parameters to maximize potency and minimize batch-to-batch variability,^112^ the inherent heterogeneity intrinsic to EVs as delivery vehicles themselves is frequently overlooked. Furthermore, while related technologies are under development,^113^^,^^114^ studies demonstrating the co-localization of functional surface molecules correlated with internal cargo at the single-vesicle level remain scarce. Despite the expression of surface molecules has been shown to guide EV fate and function,^115^ the relationship between drug encapsulation efficiency and either vesicle size or the density of target surface markers remains largely undefined. To address heterogeneity challenges impeding the clinical translation of EVs, researchers have proposed three foundational pillars.^116^ First, implementing complementary single-vesicle analytical methods, such as nanoparticle tracking analysis (NTA), resistive pulse sensing (RPS), and interferometric reflection-fluorescence microscopy, allows high-resolution characterization of EV heterogeneity. Employing calibration techniques, like utilizing NIST reference materials, mitigates bias and ensures assessment of size and molecular distribution at the single-particle level, facilitating the identification of critical subpopulations. Second, establishing standardized functional assays is essential to compare the functional disparities among distinct EV subpopulations within specific application contexts. This entails defining mechanisms of action (MOA), utilizing multiplexed complementary assays (e.g., cellular uptake and immunomodulation tests), implementing controlled dosing (e.g., based on particle number or protein mass), and incorporating control experiments (e.g., genetic knockouts or functional blocking antibodies) to verify subpopulation-specific functionality using quantifiable metrics. Finally, leveraging artificial nanovesicles (ANVs) as benchmarks or surrogates offers a strategy to overcome the heterogeneity and low yields associated with natural EVs. By engineering ANVs with controlled dimensions and molecular compositions, such as synthetic liposomes mimicking EV membrane proteins, key attributes can be standardized, although current ANV synthesis faces challenges in accurately replicating complex molecular assemblies. Consequently, the development of high-resolution isolation technologies, like microfluidic devices, is needed to enrich target subpopulations. Ultimately, coordinated optimization of analytical strategies, validation methods, and synthetic approaches can transform EV heterogeneity from a significant obstacle into a foundation for precision-targeted therapeutics.

Current storage technologies for EVs exhibit significant limitations, potentially compromising their concentration, composition, and structural integrity through multiple mechanisms. These compromises can subsequently affect research reproducibility and clinical efficacy. Conventional short-term storage at 4°C, while maintaining basic stability, is susceptible to selective loss of small-diameter EVs due to adsorption onto tube walls. This artifact can lead to misleadingly elevated concentration measurements. Concurrently, such storage conditions can induce RNA leakage, protein degradation, and conformational alterations of surface markers (e.g., HSP70 and CD63/CD9).^117^^,^^118^ For long-term preservation, the widely adopted protocol of freezing at −80°C (as recommended by guidelines like MISEV2014) is also documented to have drawbacks. Ice crystal formation during freezing disrupts the integrity of the phospholipid bilayer, promoting cargo leakage and reducing the surface Zeta potential. Furthermore, prolonged cryostorage induces shifts in particle size distribution, aggregation/fusion of particles, and attenuation in the expression levels of characteristic proteins (e.g., Alix and TSG101). Critically, repeated freeze-thaw cycles significantly exacerbate these detrimental effects.^119^^,^^120^^,^^121^ At present, no storage methodology can fully preserve the native biological characteristics and functions of EVs. Consequently, the preferential use of freshly isolated samples remains the optimal choice for ensuring result reliability in practical applications.

Dose determination and safety assessment pose equally complex challenges. The *in vivo* metabolic profile of EVs remains incompletely elucidated, as their clearance kinetics and biodistribution characteristics are influenced by a combination of administration route, cellular origin, and disease pathology. Studies indicate that systemic administration of EVs can exert diverse biological effects on recipient cells: certain EVs may trigger apoptosis or immunomodulation, whereas others promote cell survival. Such off-target biological effects have the potential to induce organ dysfunction.^2^ While local administration can mitigate systemic impacts, it shares the fundamental limitation of rapid *in vivo* metabolism.^122^ Furthermore, dose selection is of critical importance; for instance, in neurodegenerative disease models, low-dose EVs demonstrate neuroprotective properties, whereas high doses paradoxically induce neuronal damage.^123^ Differential biological activity stemming from EV source, optimization of administration pathways, and the specific requirements of the therapeutic target must all be rigorously integrated into the dosing framework.^124^

As biological products, EV-based therapies require rigorous regulatory approval by agencies such as the US Food and Drug Administration (FDA) or the European Medicines Agency (EMA). However, the current regulatory landscape for EV therapeutics remains inadequately defined. During the approval process, regulatory agencies typically mandate the submission of comprehensive data covering manufacturing processes, product stability, safety, and efficacy. Owing to inherent technological limitations, achieving product standardization presents significant difficulties, thereby impeding the establishment of unified regulatory criteria. Consequently, this lack of harmonized standards significantly constrains the initiation and conduct of related clinical trials.

Collectively, translating the therapeutic application of EVs in antiviral contexts necessitates coordinated breakthroughs across several domains. Future research must prioritize developing standardized manufacturing protocols, refining high-purity isolation techniques, and implementing stability optimization strategies. Concurrently, a comprehensive understanding of EV *in vivo* metabolic mechanisms and quantitative dose-response relationships, alongside the establishment of robust regulatory frameworks, is imperative. Promoting multi-dimensional technology integration and clinical validation can overcome existing barriers and fully realize the potential of EVs as next-generation delivery platforms.

## Acknowledgments

This review was funded by the 10.13039/501100004608Natural Science Foundation of Jiangsu Province (No. BK20241824), the Suzhou Municipal Science and Technology Development Plan (No. SKYD2023002), the open project of Jiangsu Key Laboratory of Medical Science and Laboratory Medicine (No. JSKLM-Z-2024-008), the Talent Research Project of Suzhou Health Talent Plan (No. GSWS2023005), and the Kunshan First People's Hospital medical health technology innovation project (No. KET DCX202401).

## Author contributions

Writing—original draft, Qing.Gao.; writing—review & editing, Qing.Gao., Y.Z., J.Z., D.S., H.X., C.B., Y.X., Qianqian.Gao., and L.M.; funding acquisition, L.M.; supervision, L.M.

## Declaration of interests

The authors declare no competing interests.
